# Initial Evaluation of a Titration Appliance for Temporary Treatment of Obstructive Sleep Apnea

**DOI:** 10.4172/2167-0277.1000101

**Published:** 2011-12-24

**Authors:** Daniel J. Levendowski, Todd Morgan, Philip Westbrook

**Affiliations:** 1 Advanced Brain Monitoring Inc., Carlsbad, USA; 2 Scripps Dental Group, Carlsbad, USA

**Keywords:** Obstructive sleep apnea, Oral appliance therapy, Titration, Mandibular repositioning, Splint

## Abstract

**Background:**

Custom oral appliances that adjustably advance the mandible provide superior outcomes when treating patients with moderate or severe sleep apnea. Custom appliances, however, are expensive, must be fitted by a dentist, and the likelihood of successful outcomes are difficult to predict. An inexpensive trial appliance, if proven efficacious, might be used to predict custom appliance outcomes or to provide temporary therapeutic benefit.

**Objective:**

The aim of this initial study was to assess the treatment efficacy of a novel titration oral appliance with that of an optimized custom appliance.

**Methods:**

Seventeen patients, treated with a custom oral appliance for at least one year, successfully completed a three-night home sleep test. The baseline obstructive sleep apnea severity was established on Night 1 with seven patients exhibiting severe, six moderate and four mild apnea/hypopnea indexes. Patients were randomly assigned to wear their custom appliance or the titration appliance on Nights 2 and 3.

**Results:**

Significant reductions in the mean overall and supine apnea indexes (p < 0.05), and the overall (p < 0.01) and supine (p < 0.05) apnea/hypopnea indexes were observed for both the titration and custom appliances. The proportion of patients who exhibited at least a 50% reduction in the overall apnea index and supine apnea/hypopnea were similar for the titration and custom appliance (~60%). The custom appliance reduced the overall apnea/hypopnea index by 50% in a greater proportion of the patients compared to the titration appliance (77% vs. 53%). The titration appliance significantly reduced the degree of hypoxic exposure across sleep disordered breathing events overall (p < 0.05) and supine (p < 0.01). Patients found their custom appliance was more comfortable than the titration appliance, but preferred the titration appliance to no therapy.

**Conclusion:**

The titration appliance may be useful in assessing oral appliance treatment efficacy. When set to 70% of maximum protrusion, the titration appliance may provide immediate, temporary therapeutic benefit.

## Introduction

Oral appliance therapy (OAT) has been demonstrated to be efficacious in the treatment of Obstructive Sleep Apnea (OSA) [1–3]. Reports suggest that long-term OAT compliance is high [4], and the dental effects are mild [5]. When properly titrated, OAT provides similar outcomes as continuous positive airway pressure in patients with mild/moderate OSA both short [6] and long-term [7]. Custom appliances, as compared to non-adjustable devices, are more likely to provide successful therapy in patients with moderate-severe OSA [8]. To fabricate a customized appliance, a dentist makes impressions and a bite registration of the patient’s upper and lower teeth. A George gauge is typically used to determine the relationship between the upper and lower dental arches in the neutral and maximum voluntary protrusive settings in order to select an initial protrusive jaw position. A dental laboratory uses the impressions to make castings, and then fabricates the custom appliance using materials that provide two or more years of useful life, articulated to the prescribed protrusive setting. Once the appliance is delivered to the patient, further adjustments are made until the mandible is optimally protruded and symptoms resolve. Depending on the mechanism used for the appliance, sub-millimeter protrusion adjustments are possible. The assessment of treatment efficacy and/or need for further adjustment is typically made with a home sleep test(s) [2,3]. Alternatively, the optimized appliance setting can be determined with the assistance of a technician during an attended sleep study [1, 8,9].

Studies have shown that not all patients with OSA respond to OAT. The primary factors that impacts therapeutic success are the appliance settings, including: protrusion, vertical separation of the dental arches, and freedom for vertical and lateral movement [10,11]. However, the ratio of the frequency of apneas to hypopneas, the positional influence on AHI severity, the neck circumference, and the body mass index can also contribute to poor outcomes [2]. Given this uncertainty about the efficacy in any given patient, and given that the cost for delivery of a custom appliance (which many patients must pay for) is between $2,000–$3,000, physicians can be reluctant to refer for a trial of OAT. Dentists, clinicians and patients would all benefit from an inexpensive trial appliance that could be easily fitted, and most importantly, could reliably predict efficacy. If proven efficacious, the appliance could also be used as a temporary appliance when required (e.g., manage OSA severity perioperatively).

This report is based on an initial evaluation of the efficacy of an inexpensive titration appliance, easily fitted in 15-minutes by any properly trained healthcare professional, for use in determining a patient’s response to oral appliance therapy or to provide immediate temporary therapy.

## Materials and Methods

Twenty subjects were enrolled after obtaining an informed consent (approved by the BioMed IRB, San Diego, CA, USA). Criteria for inclusion included: 1) Completion of a baseline ARESTM (ARES Medical, Carlsbad, CA) home sleep test with an apnea/hypopnea index greater than 10 events per hour, 2) Being fitted with either a Tap III (Airway Labs, Dallas, Tx) or Herbst (Great Lakes Orthodontics, Tonawanda, New York) custom mandibular repositioning device, 3) Completed multi-night home sleep tests used to select the optimal protrusion setting, and 4) Continuously wearing the same custom appliance for greater than one year.

Subjects were expected to complete a three-night ARES sleep study with a minimum of five hours of recording time per night. One subject was inadvertently studied and then dropped because he had replaced the custom appliance required for the study. Two subjects were unable to successfully complete the night’s sleep with the titration appliance. Those included for analysis were12 males and five females, with a mean age of 58 + 9.0 SD (range 34 to 69) years, BMI of 28 + 4.4 SD (range 19 to 37) kg/m2, and neck circumference of 41 + 4.4 SD (range 34 to 48) cm.

The baseline obstructive sleep apnea severity was established on Night 1 when no appliance was worn. To minimize risk to the patient (given the OSA severity of those recruited), the protocol did not include a wash-out period to eliminate possible carry-over benefit from treatment. For Nights 2 and 3, subjects were randomly assigned to wear either their custom appliance or a titration appliance set to 70% of maximum voluntary protrusion. Ten subjects who successfully completed the overnight studies wore the Tap III and seven wore the Herbst appliance. After the three night study, patients completed a survey to compare the comfort and sleep quality with custom vs. titration appliances vs. no appliance.

Sleep disordered breathing severity outcome measures from the sleep studies were derived using the ARES auto-scoring algorithms. The description and accuracy of this device as compared to laboratory polysomnography has been previously reported [12–16]. The primary outcome measures stratified by night included the apnea index (i.e., > 10-second cessation in airflow) and apnea/hypopnea index with a 4% oxyhemoglobin desaturation (AHI-4%) by and across positions, percentage of sleep time with snoring greater than 30, 40 or 50 dB, and the average reduction in SpO2 across all events with a 50% reduction and recovery in tidal volume.

**Figure 1:**
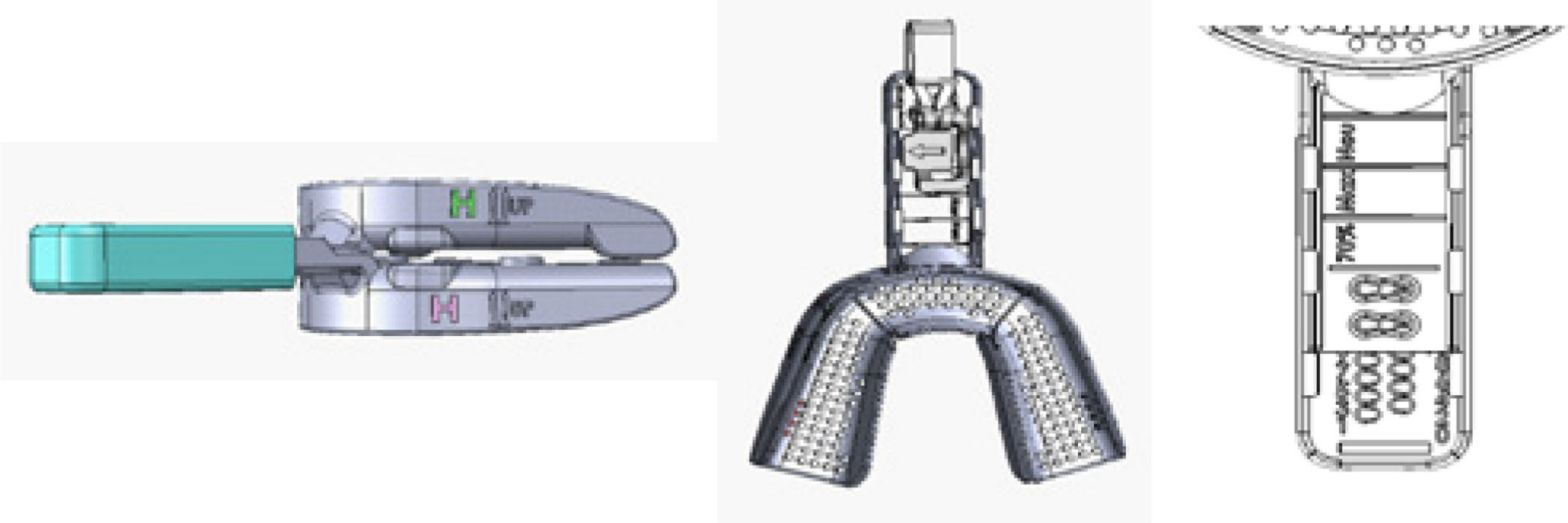
Temporary appliance with a) side view with lock covered, b) top view with lock in place and c) adjustment numbers locking holes, and location to record the titration measurements.

The titration appliance used in this study was the Apnea Guard^®^ (Advanced Brain Monitoring, Carlsbad, CA) (Figure 1). The upper and lower trays expand to accommodate a full range of arch widths. The tethered locking mechanism provides one millimeter adjustments across protrusive ranges from 8 mm retrognathic to 18 mm prognathic. Standard dental impression material is used to provide denture retention. The size (High) was used in this study provided 8 mm of anterior and 6 mm of posterior vertical dimension of occlusion. Posterior posts provide bite support for patients with bruxism. Prototype appliances were used in this study; the device has been subsequently approved for up to 30-nights of use.

**Figure 2:**
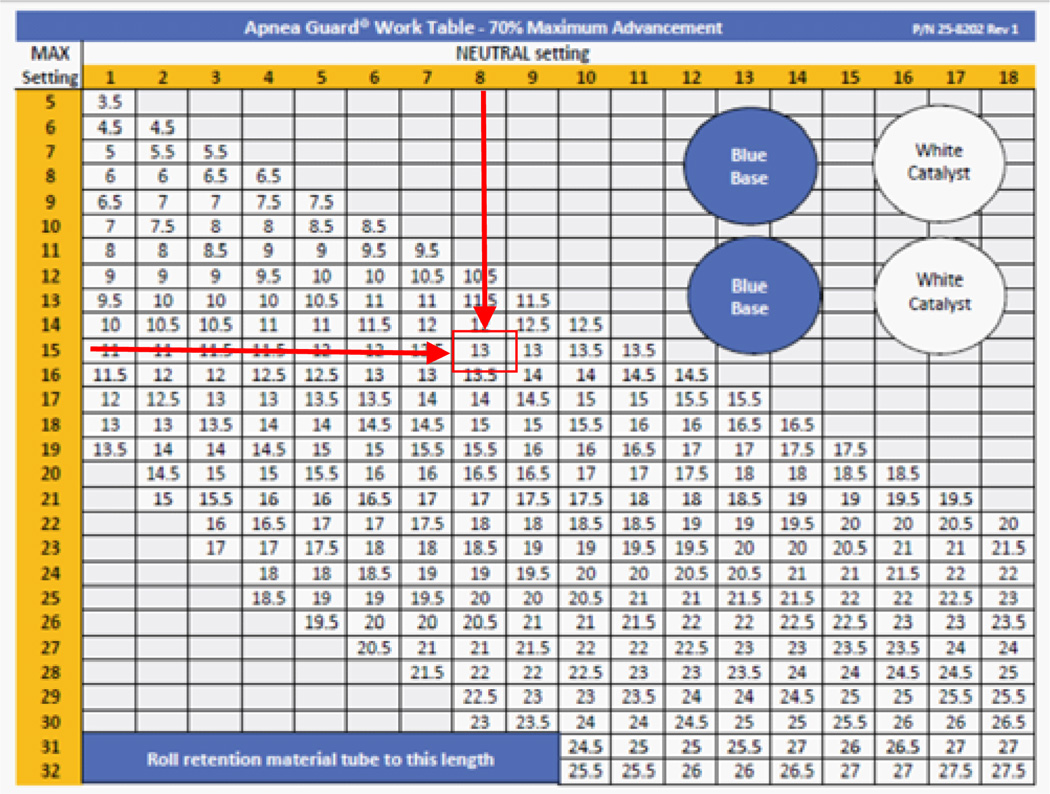
Work table used to determine 70% maximum protrusion based on neutral (column) and maximum voluntary protrusive (row) settings.

A systematic approach was employed to measure the optimal amount of retention material and prepare the appliance. A specific amount of retention material is measured for the upper and lower trays. Beginning with the lower tray, the teeth are fitted with the retention material. A recommended sequence for fitting the lower and upper trays ensures the retention material provides protection for all of the teeth. Once the retention material is fitted, the neutral and maximum voluntary protrusions are determined with the titration appliance inserted. The neutral (column) and maximum (row) values are applied to the work table (Figure 2) to derive the setting that corresponds to a 70% of maximum protrusion. The work table presents protrusion settings in 0.5 mm increments to accurately reflect 70% protrusion; however the appliance provides allows 1 mm incremental adjustments. In this study, protrusions setting were rounded up to the nearest integer value because patients were accustomed sleeping with an advanced mandible position. The appliance is designed so the neutral, maximum and 70% settings can be recorded on the device for future reference.

To assess how closely the 70% protrusion of the temporary appliance matched the protrusive setting for the custom appliance, marks were made on the upper and lower trays at the anterior aspect of the first molar. Calipers were used to measure the distance between the marks. The neutral and maximum voluntary protrusion were measured with both the titration appliance and a George gauge for subsequent comparison. Measures were repeated three times to assess reliability.

Given the expected directional change resulting from initiation of therapy, one-tailed t-tests were used to assess statistically significant changes in OSA severity between baseline vs. the titration or custom oral appliance.

## Results

No differences were observed in the mean overall, supine or non-supine valid sleep times. A minimum of 18 minutes of valid sleep time was required to derive positional OSA severity values. Two patients slept exclusively supine during the baseline test, and one patient slept supine across all three nights. Insufficient supine time was observed in two patients with the titration appliance and one patient with custom appliance.

**Figure 3:**
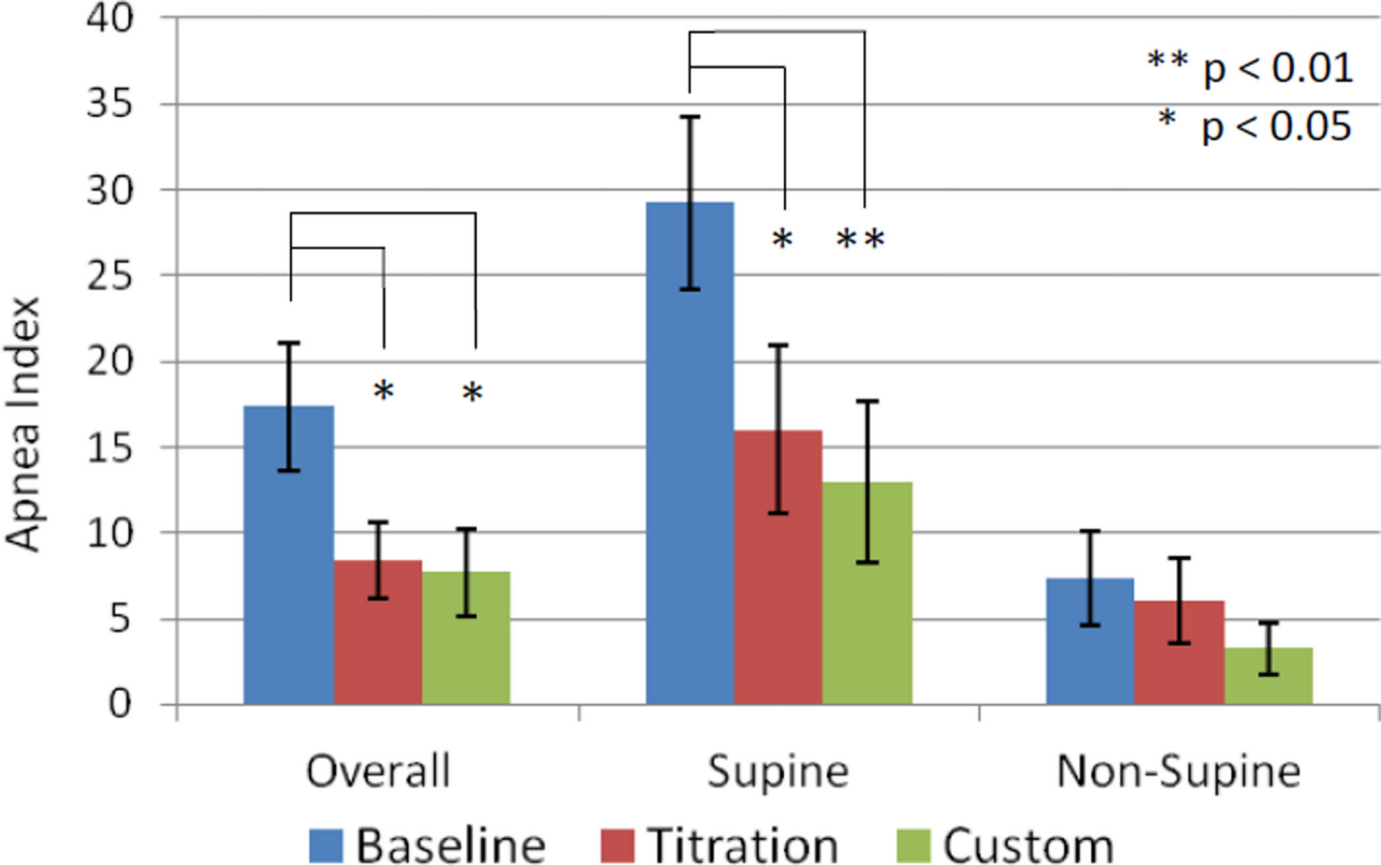
Mean + standard error for the overall, supine and non-supine apnea index at baseline and with the titration and custom oral appliances.

The mean overall, supine and non-supine apnea index values are presented in (Figure 3) at baseline and with the titration and custom appliance. Seven patients exhibited severe (AHI > 30), six moderate (AHI 15 – 29) and four mild (AHI < 15) OSA. Changes in the mean overall values were significant for both the titration and custom appliances (p = 0.022 and 0.027 respectively). The minimum estimated reduction that would be expected based on the low confidence interval (L-CI) was 2 apneas/hr for the titration appliance and 1 apnea/hr for the custom appliance. Significant changes were also observed in the supine apnea index for the titration and custom appliances (p = 0.035 and 0.006, L-CI = 1 and 6 events/hr, respectively). The percentage of patients with a baseline overall apnea index > 5 that showed at least a 50% reduction was 60% for the titration appliance (9 of 15) and 64% for the custom appliance (9 of 14).

**Figure 4:**
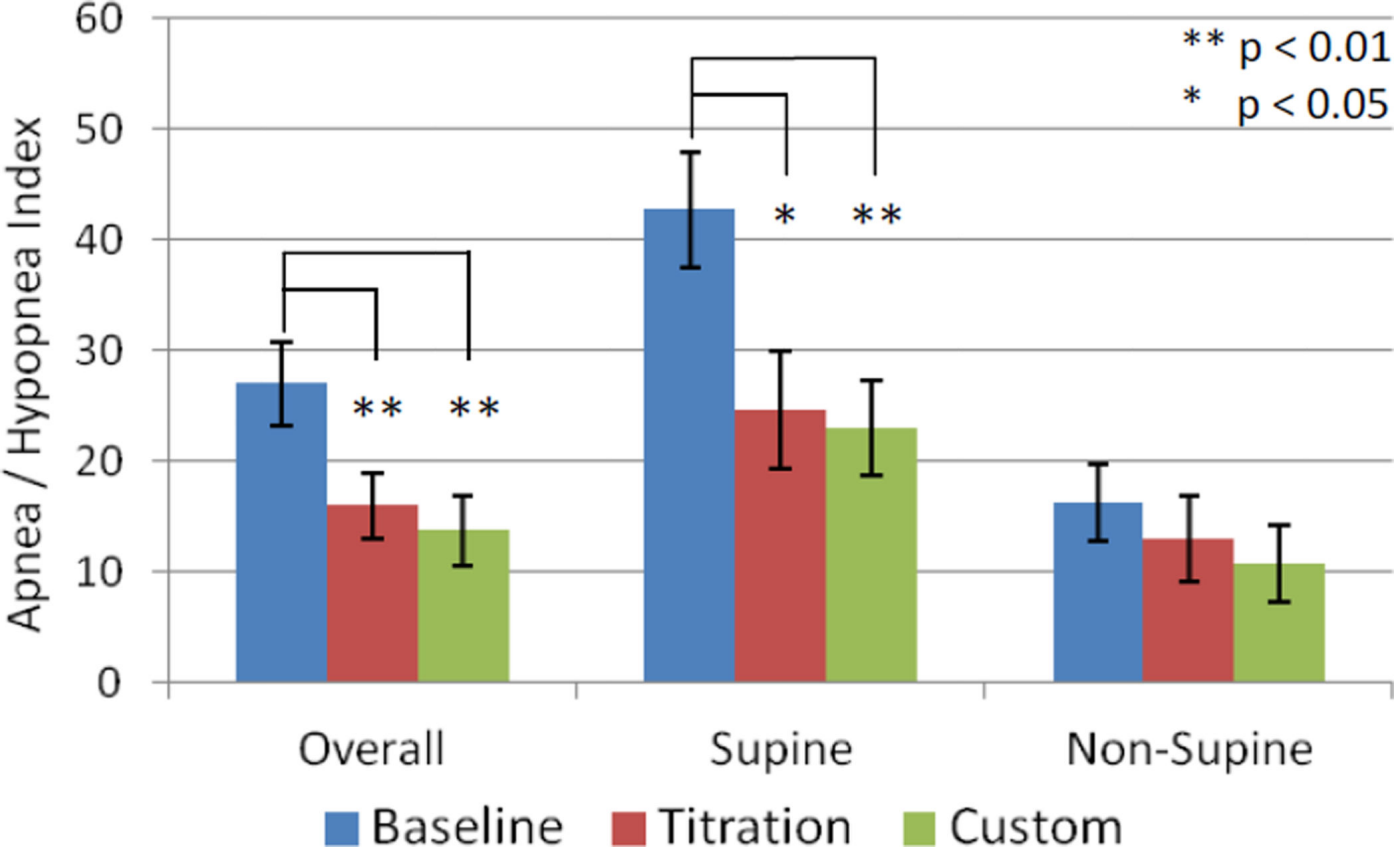
Mean + standard error for the overall, supine and non-supine apnea/hypopnea index at baseline and with the titration and custom oral appliances.

**Figure 5:**
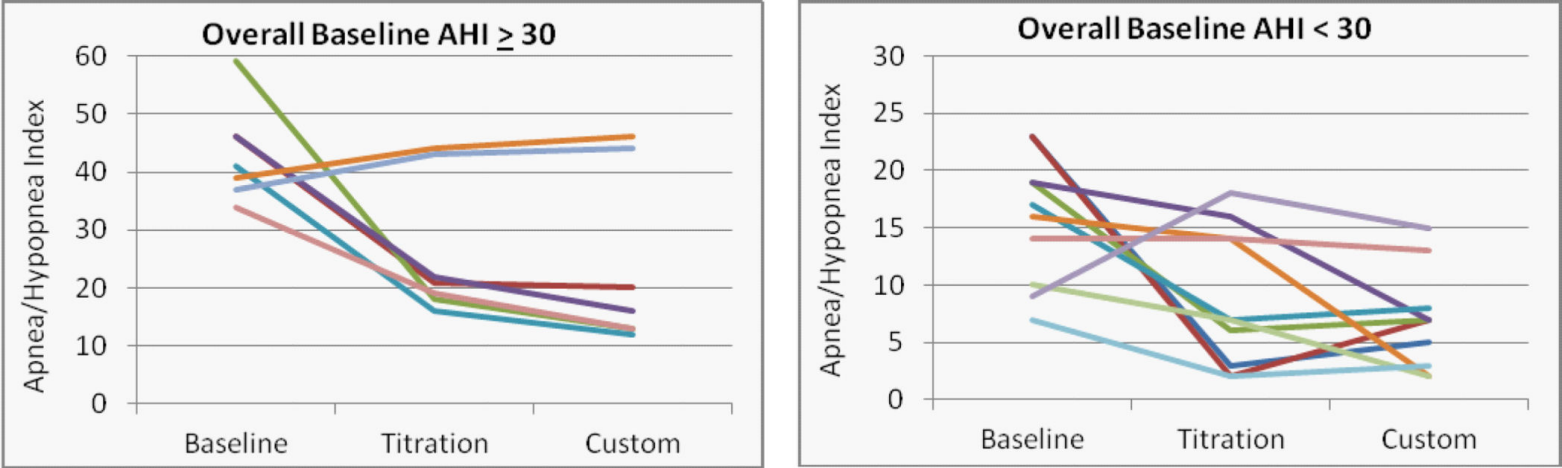
Changes in the overall apnea/hypopnea index with the titration and custom appliance in patients with a) severe, and b) mild or moderate OSA.

**Figure 6:**
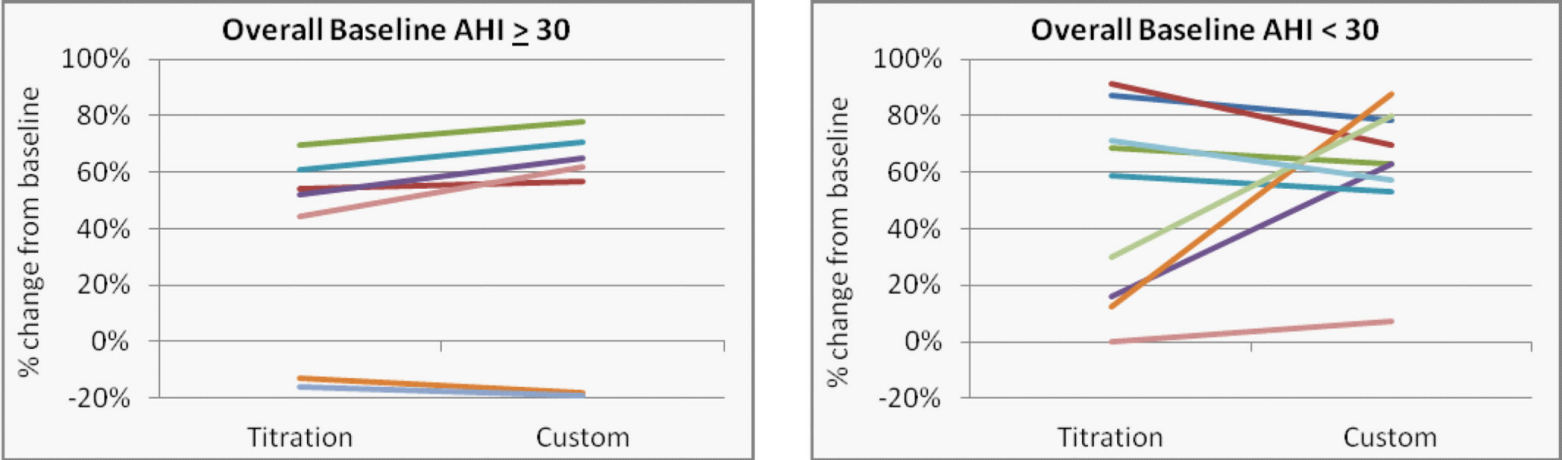
Percent change from baseline in the overall apnea/hypopnea index with the titration or custom appliance in patients with a) severe, and b) mild or moderate OSA with outlier that changed −100% and – 67% not shown (AHI - baseline = 9, titration = 18, and custom = 15).

**Figure 7:**
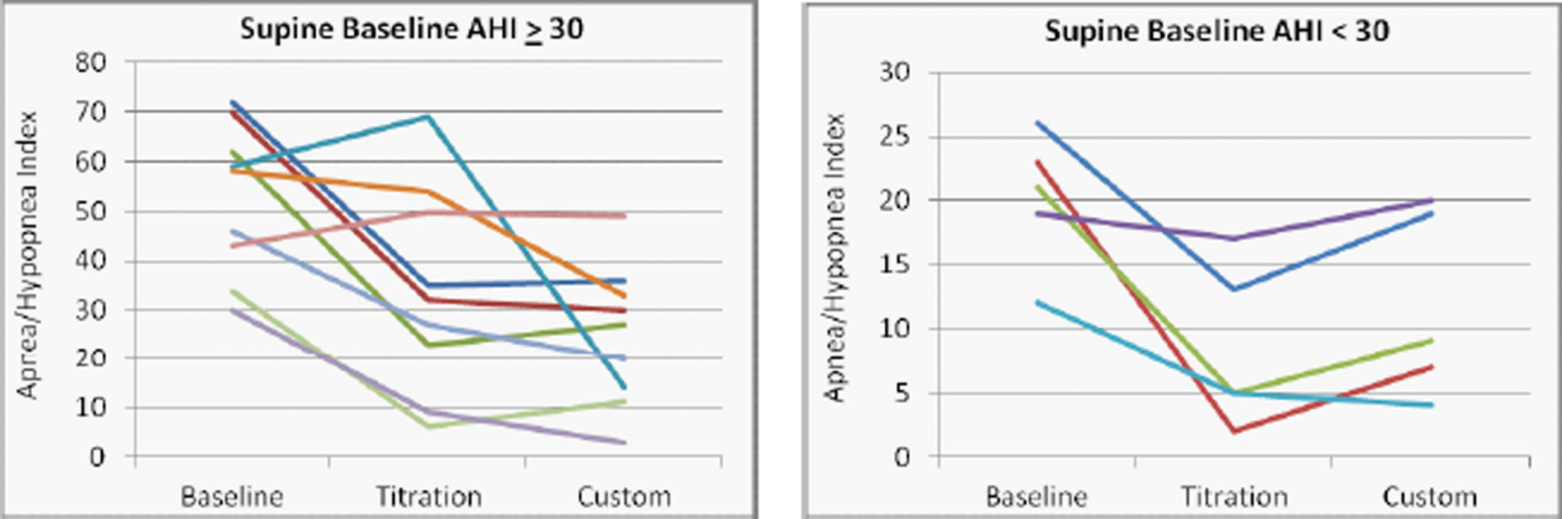
Changes in the supine apnea/hypopnea index with the titration or custom appliance in patients with a) severe, and b) mild or moderate OSA.

**Figure 8:**
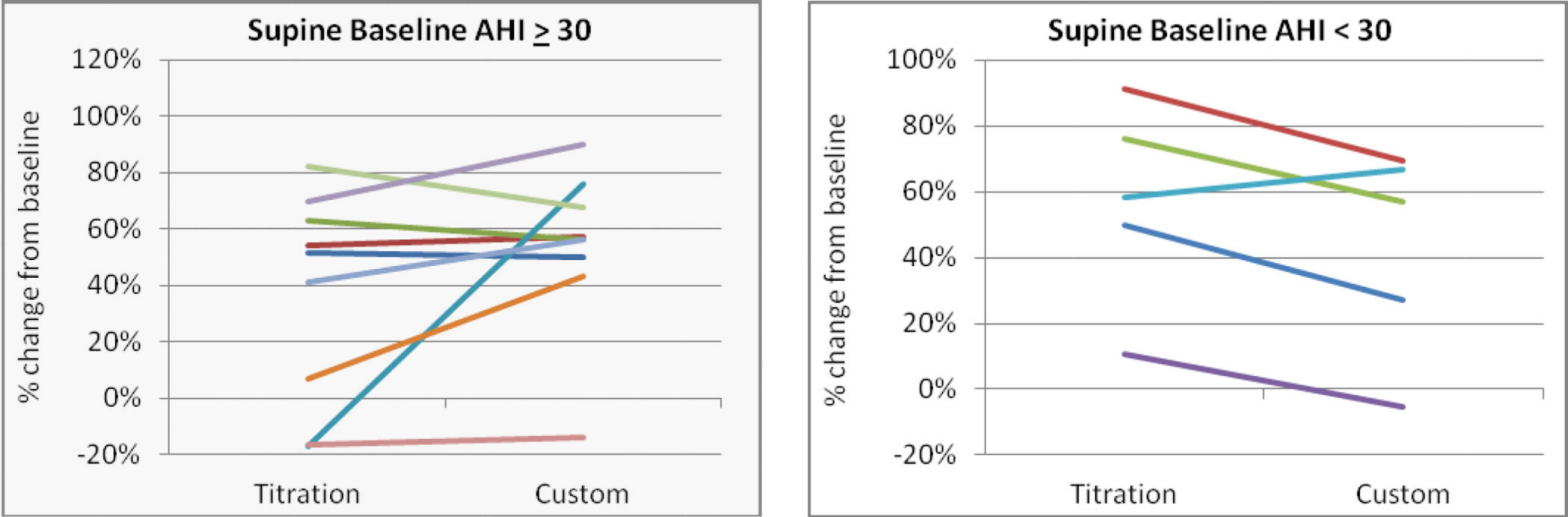
Percent change from baseline in the supine apnea/hypopnea index with the titration or custom appliance in patients with a) severe, and b) mild or moderate OSA.

**Figure 9:**
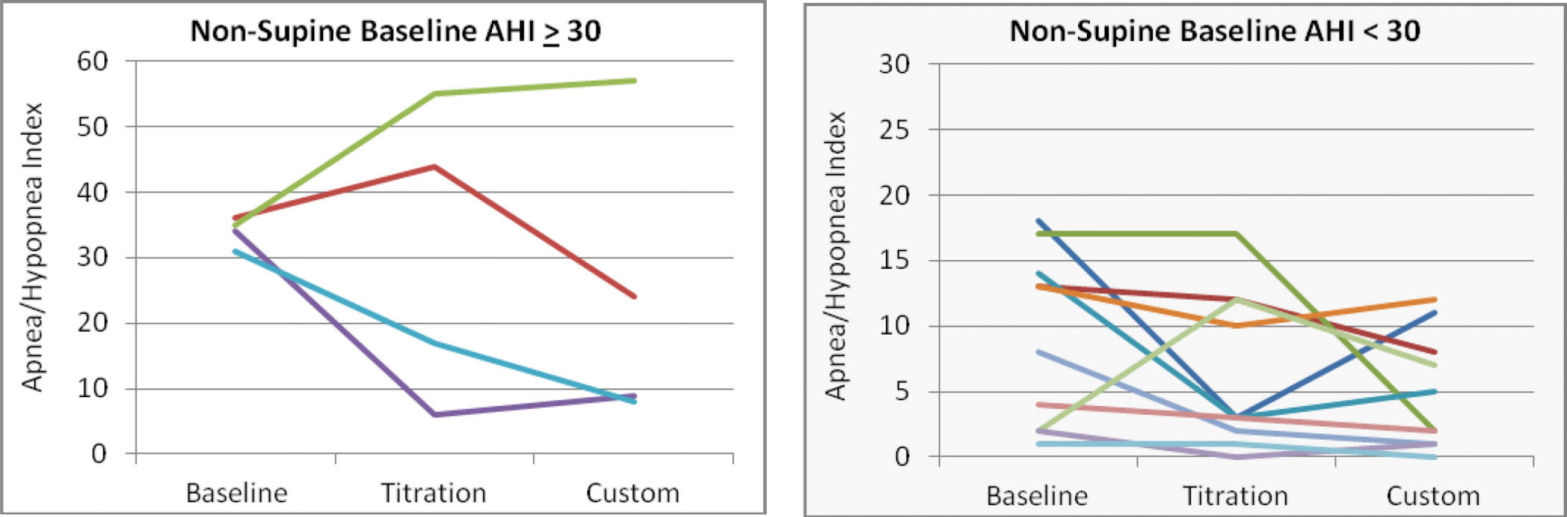
Changes in the non-supine apnea/hypopnea index with the titration or custom appliance in patients with a) severe, and b) mild or moderate OSA.

The mean overall, supine and non-supine apnea/hypopnea index values are presented in (Figure 4) at baseline and with the titration and custom appliances. Significant reductions in the mean values were observed for both the titration and custom appliances overall (p = 0.009 and 0.005, L-CI = 4 and 5 events/hr) and supine (p = 0.027 and 0.002, L-CI = 4 and 9 events/hr). Changes from baseline for individual cases were stratified by severe (AHI > 30) and moderate/mild OSA and presented for the overall AHI (Figure 5), supine AHI (Figure 7) and non-supine AHI (Figure 9). At least a 50% reduction in overall AHI was observed in 53% (9 of 17) of the patients with the titration appliance and 77% of those with the custom appliance (13 of 17). Comparisons of the percent change stratified by OSA severity are presented in (Figure 6). The proportion of patients with at least a 50% reduction in a measurable supine AHI was 67% (10 of 15) with the titration appliance and 63% (10 of 16) with the custom appliances. Differences in the percentage change in supine AHI from baseline are presented in (Figure 8).

**Figure 10:**
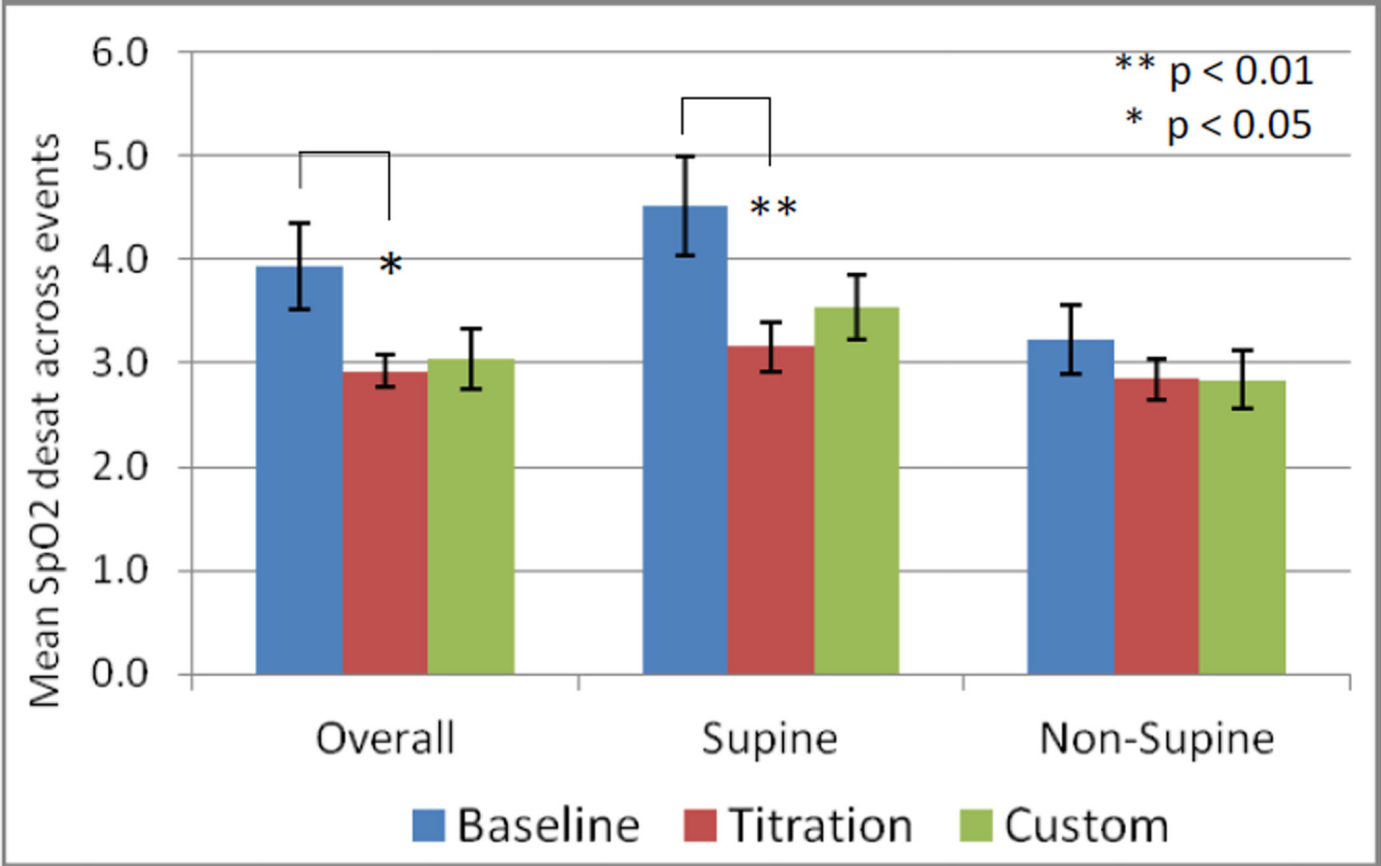
Mean + standard error for the overall, supine and non-supine SpO2 desaturation across events with a 50% reduction in tidal volume.

**Figure 11:**
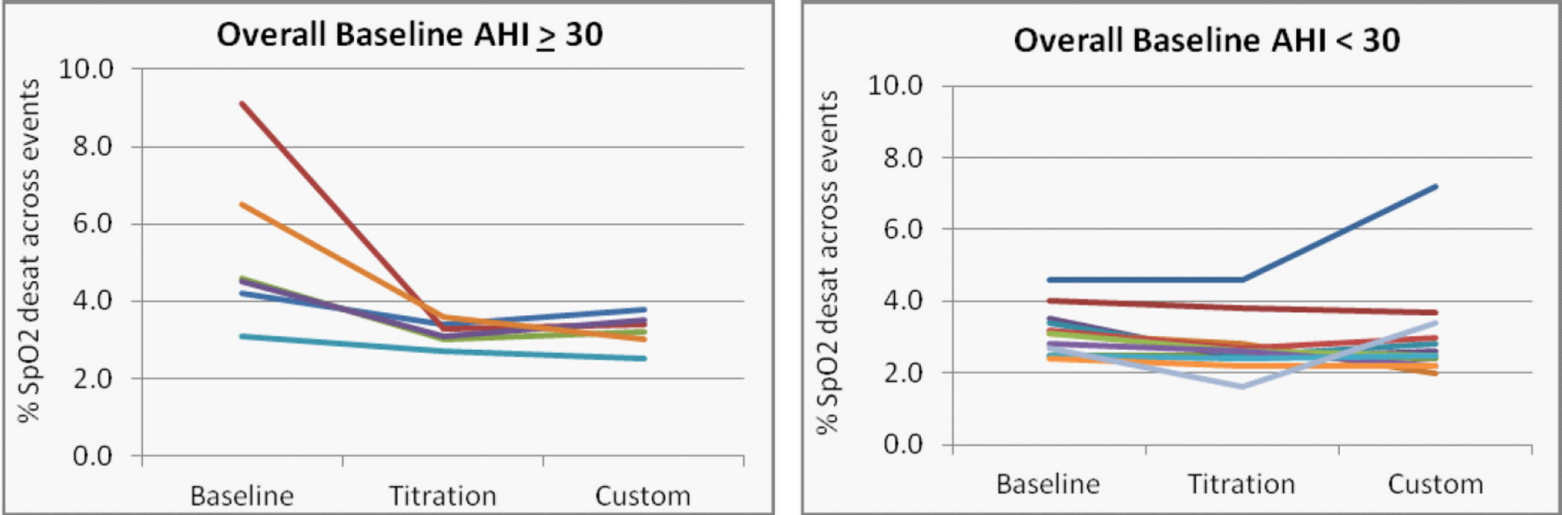
Changes in the SpO2 desaturation across all events with a 50% reduction in tidal volume in patients with a) severe, and b) mild or moderate OSA.

**Figure 12:**
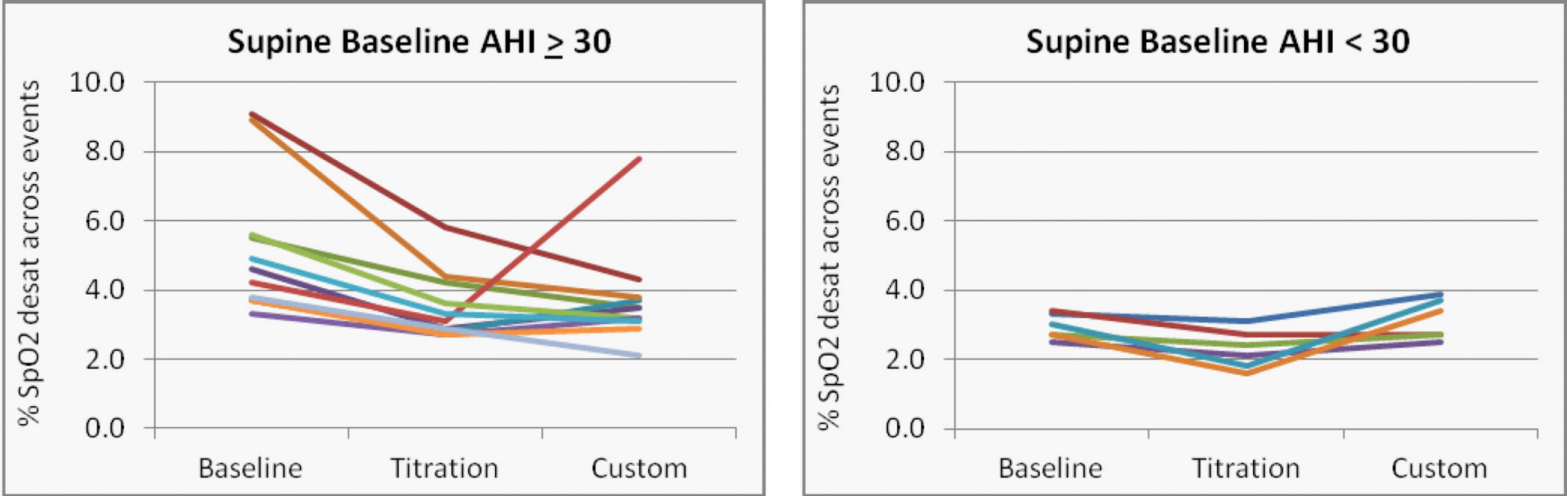
Changes in the SpO2 desaturation across supine events with a 50% reduction in tidal volume in patients with a) severe, and b) mild or moderate OSA.

Significant reductions in SpO2 reductions across sleep disordered breathing events were obtained with the titration appliance overall and supine (Figure 10) (p=0.014 and 0.006, L-CI = 0.3 and 0.5 % reduction in SpO2). The effect of the custom appliance on the reduction in SpO2 desaturation was close to significant overall and supine (p = 0.058 and 0.055). Individual cases, stratified by OSA severity overall and in the supine position are presented in (Figures 11 and 12).

The percentage of time snoring greater than 30, 40 or 50 dB was not significantly impacted by appliance and position. Twelve patients snored above 50 dB for greater than 5% of the night at baseline; the titration appliance and custom appliances reduced the snoring by at least 50% in seven and eight cases (58% and 67%), respectively. The titration appliance reduce the percentage of time snoring greater than 40 dB at baseline in 44% (7 of 16) of the cases while the custom appliance reduced snoring in 56% (9 of 17) of the cases.

Use of titration appliance to determine 70% protrusion resulted in 79% of the patients being advanced exactly the same (11/19) or 1 mm less (4/19) than the custom appliance. One patient was advanced 2 mm less, another 2 mm more than the custom appliance but both had similar outcomes. One patient was advanced 4 mm less than the custom appliance but neither appliance provided therapeutic benefit. Another patient was advanced 4 mm less than the custom appliance, and this setting may have contributed to the titration appliance being 18% less efficacious.

Estimations of neutral and the maximum voluntary protrusion were least reliable in the first of the three trials. Rejection of the first measure and averaging of the next two trials was superior to averaging across the three trials. Variability was more apparent when determining the neutral position with the titration appliance and the maximum protrusion setting with the George gauge. In 74% of the cases (14/19), the George gauge measured a greater voluntary range than the titration appliance, possibly explained by differences in the VDO during measurement (i.e., George gauge = 4 mm and the titration appliance = 8 mm). Two outliers, whereby the range of protrusion was 2 and 4 mm greater with the titration appliance vs. the George gauge, did not appear to impact efficacy.

Seventeen of the 19 patients who wore the titration appliance completed the survey. The six survey questions are presented in Table 1 in conjunction with the median responses to each question. Responses to each question included: strongly agree, agree, neither agree nor disagree, disagree, or strongly disagree. Table 2 identifies features of the titration appliance reported by patients as contributing to less comfort or poorer quality sleep as compared to their custom appliance.

## Discussion

This is the initial report pertaining to the efficacy and reliability of a previously fabricated custom appliance set to an optimized protrusion and the titration appliance set to 70% protrusion. Both appliances performed well with significant reductions in the mean overall and supine apnea and apnea/hypopnea indexes observed. Seventy-seven percent of patients exhibited efficacious outcomes with the custom appliance, based on a 50% reduction in AHI, consistent with previous reports. In three cases, the titration appliance proved inferior to the custom appliance. In two of the three cases, notes from the patient indicated the titration appliance may have either been removed or did not function properly during the night. A one-night baseline (without a wash out period) combined with a single night with 5-hours of sleep time per appliance may have contributed to measurement variability.

Although oral appliances are commonly prescribed to reduce snoring, mean reductions in snoring were not observed in this data set. It is likely that the number of patients with high baseline apnea indexes prior to the splinting of the airway contributed to this finding, given the conversion of apneas to hypopneas increases the percentage of sleep time with loud snoring.

The results from this study while promising should be interpreted with caution given the small sample size and inclusion of patients previously treated with a custom appliance. The findings point to the need for prospective exploration of a number of topics related to both oral appliance therapy in general and the titration appliance specifically.

Case reports suggest airway patency adapts slightly after initiation of oral appliance therapy at 60% protrusion. This factor contributes to the need for subsequent advance. The assumption that 70% protrusion for the titration appliance would be close to optimal was confirmed by the fact that the mandibular advancement of the titration and custom appliances were essentially the same in over 80% of the cases. The study design did not address the concern that initiation of therapy at 70% protrusion vs. the traditional 60% may increase the level of initial discomfort in patients who have not previously undergone oral appliance therapy. Future studies are planned to compare the impact of initiating therapy at 60% vs.70% protrusion on the number of days patients report morning muscular discomfort. The timing of when to perform the efficacy outcome study also needs to be explored. Sleep study results obtained during the first night of therapy may be useful in identifying patients who respond to oral appliance therapy. It is unclear whether these initial results can also establish the appliance has been optimally set.

The two custom appliances used in this study employ different mechanics to position the mandible. One of the appliances provided for subtle adjustment of the VDO while the other is fixed. A lack of control over the vertical dimensions of occlusion (VDO) settings of the custom appliances did not appear to contribute to a systemic bias, although the sample sizes were too small for statistical comparison. The investigators believe that the 8 mm VDO of the titration appliance was greater than the VDO settings of the custom appliances in most cases. Although the impact of VDO on oral appliance outcomes is unclear, preliminary evidence suggests males and females respond differently [17]. Increasing the VDO provides more room in the oral cavity for large tongues to advance. It is possible that the increased VDO explains why the titration appliance was slight less effective in reducing the number of apneas and hypopneas (primarily in the non-supine position), while simultaneously providing superior management of hypoxemia across all sleep disordered breathing events with a 50% decrease in tidal volume. Subsequent to this study titration appliances were developed with 5.5(low) and 6.5 mm (medium) VDO to complement the 8.0 mm (high) VDO appliance. A study is underway to compare differences in outcomes when patients wear the low, medium and high VDO, all advanced to 70% protrusion.

The titration appliance could be used in additional research to investigate how to enhance OSA therapies. For example, if the VDO or protrusion settings that optimize oral appliance therapy outcomes are different for the supine vs. non-supine position, outcomes could be optimized by adjusting the oral appliance for the non-supine position and using it combination with position therapy. The titration appliance could be used to assess whether acceptance of nasal EPAP is enhanced by mandibular advancement and/or whether an oral appliance can be used as an alternative to a chin strip to reduce mouth breathing. The benefit of combined OAT and surgery could be inexpensively assessed by ear, nose and throat physicians prior to referral for a custom appliance.

The titration appliance is limited to 30-nights of use based, in part, on the assumption that patients with no contra-indications can safely wear it for a short duration. The 30-day window cleared by FDA provides time for a patient to be fitted and undergo a titration study initiated by their physician and continue therapy until a dentist can deliver a custom appliance. Alternatively, if the appliance is used to manage OSA perioperatively, sufficient time is provided for the patient to recover from surgery, receive a consult and be diagnosed and treated. In both of these examples, non-dental personnel are expected to fit and set the titration appliance. Studies are underway to demonstrate that side effects are limited with use of the dental history and consent form, patient instructions and training materials developed for fitting the appliance by sleep center or hospital personnel staff.

Patients found the custom appliance more comfortable, but preferred the titration appliance to no therapy. To avoid a negative bias toward OAT developing during the trial period, patients need to be educated that custom appliances are more comfortable and that excessive salivation during sleep will subside. Additional studies are needed to assess compliance in patients who have not previously worn an oral appliance, especially if the titration appliance were to be used for management of perioperative OSA severity.

Studies are underway to demonstrate that the VDO size and protrusion setting of the titration appliance combined with extracted retention material used for the bite registration allows the demonstrated optimal relationship between the upper and lower dental arch to be articulated into the custom appliance. This approach could reduce the effort required by dentists and patients to adjust and test the custom appliance. It would also increase consistency of outcomes when custom appliances are fitted by less experienced dentists. Patients could continue to using the titration appliance when refitted with a second set of retention material until the custom appliance is delivered.

## Conclusions

This study suggests that a titration appliance that provides that capability for 1 mm incremental mandibular advancement can be used to assess oral appliance treatment efficacy. Alternatively the appliance, when set to 70% of maximum protrusion may provide immediate, temporary therapy for OSA. Future studies are needed to confirm these findings.

## Figures and Tables

**Table 1:** Questions and responses to Comfort and Sleep Quality Survey.

Survey Question	Median response
1. The comfort of my custom appliance was similar to the titration appliance	Disagree
2. My custom appliance was more comfortable than the titration appliance	Agree
3. The quality of my sleep when I wore my custom appliance was similar to the titration appliance	Neither agree or disagree
4. My sleep quality was better when I wore my custom appliance compared to the titration appliance	Agree
5. The quality of my sleep when I was NOT wearing either appliance was similar to when I wore my custom appliance	Disagree
6. The quality of my sleep when I was NOT wearing either appliance was similar to when I wore the titration appliance	Disagree

**Table 2:** Features that made titration appliance less comfortable than the custom appliance.

Complaint	n (%)	Complaint	n (%)
Increased nocturnal drooling	5 (26)	Did not retain as well to teeth	3 (16)
Rubbed against gums or tongue	5 (26)	Bulky	2 (11)
Could not maintain a lip seal	3 (16)	Restricted jaw movement	2 (11)
